# Components of antenatal care received by women in fishing communities on Lake Victoria, Uganda; a cross sectional survey

**DOI:** 10.1186/s12913-020-05739-9

**Published:** 2020-09-29

**Authors:** Ali Ssetaala, Joan Nabawanuka, Gideon Matovu, Nusula Nakiragga, Judith Namugga, Phiona Nalubega, Henry Lutalo Kaluuma, Kundai Chinyenze, Katrina Perehudoff, Kristien Michielsen, Bernard Bagaya, Matt Price, Noah Kiwanuka, Olivier Degomme

**Affiliations:** 1 UVRI-IAVI HIV Vaccine Program, Entebbe, Uganda; 2 Ghent University International Centre for Reproductive Health, Ghent, Belgium; 3 IAVI, New York, NY USA; 4 Makerere University College of Health Sciences, Kampala, Uganda; 5 Department of Epidemiology and Biostatistics, University of California at San Francisco, San Francisco, CA USA

**Keywords:** Antenatal, care, Components, Women, Islands, Fishing, Community, Uganda

## Abstract

**Background:**

Uganda has one of the highest maternal deaths at a ratio of 336 per 100,000 live births. As Uganda strives to achieve sustainable development goals, appropriate antenatal care is key to reduction of maternal mortality. We explored women’s reported receipt of seven of the Uganda guidelines components of antenatal care, and associated factors in hard to reach Lake Victoria island fishing communities of Kalangala district.

**Methods:**

A cross sectional survey among 486 consenting women aged 15–49 years, who were pregnant at any time in the past 6 months was conducted in 6 island fishing communities of Kalangala district, Uganda, during January–May 2018. Interviewer administered questionnaires, were used to collect data on socio-demographics and receipt of seven of the Uganda guidelines components of antenatal care. Regression modeling was used to determine factors associated with receipt of all seven components.

**Results:**

Over three fifths (65.0%) had at least one ANC visit during current or most recent pregnancy. Fewer than a quarter of women who reported attending care at least four times received all seven ANC components [(23.6%), *P* < 0.05]. Women who reported receipt of ANC from the mainland were twice as likely to have received all seven components as those who received care from islands (aOR = 1.8; 95% CI:0.9–3.7). Receipt of care from a doctor was associated with thrice likelihood of receiving all components relative to ANC by a midwife or nurse (aOR = 3.2; 95% CI:1.1–9.1).

**Conclusions:**

We observed that the delivery of antenatal care components per Ugandan guidelines is poor in these communities. Cost effective endeavors to improve components of antenatal care received by women are urgently needed. Task shifting some components of ANC to community health workers may improve care in these island communities.

**Trial registration:**

PACTR201903906459874 (Retrospectively registered).

## Background

Uganda has one of the highest maternal deaths with a mortality ratio of 336 per 100,000 live births, mostly in{FormattingCitation} rural, resource limited, hard-to-reach settings [1]. Maternal deaths are often from hemorrhage, hypertension, sepsis, unsafe abortions and other indirect causes like malnutrition, HIV, malaria, poor utilization of health services, poor quality care, critical shortages of skilled attendants and socio-cultural, economic factors [2–4].

Components of antenatal care (ANC), involving investigations and interventions by a Midwife, Nurse or Doctor on a pregnant woman are important in averting maternal deaths [5, 6]. The current Uganda clinical guidelines recommend at least four goal oriented ANC visits, far below the current WHO recommendations of at least eight contacts for a positive pregnancy experience and reduction of perinatal mortality [7, 8]. The ANC visits should provide components of care that include blood pressure measurement, fetal growth monitoring, urine testing, iron-folic acid supplementation, tetanus vaccination, at least three doses of Intermittent Preventive Treatment with Sulphadoxine/pyrimethamine (IPTp), deworming after the first trimester, blood group typing if not done previously, HIV and syphilis testing [7]. The 2016 Uganda demographic and Health survey indicates that almost all women from island communities with a childbirth during the preceding 5 years, had at least one ANC visit [1]. However, ANC visits attendance per se doesn’t directly translate into receipt of care components, yet these impact on the quality process, affecting subsequent visits, cost of care, skilled birth attendance and eventual prevention of maternal-child deaths [9–12].

Per the Avedis Donabedian model for quality of care [13], ANC components may be an important element in assessing quality, especially in situations where there is a barely adequate availability of structural inputs. Blood pressure measurement aids the diagnosis, prevention, and management of hypertensive conditions of pregnancy including chronic hypertension, gestational hypertension, preeclampsia, eclampsia, and preeclampsia superimposed on chronic hypertension [14]. Urine testing is key in the early diagnosis, prevention and management of urinary tract infections, gestational diabetes, preterm labor, low birth weight and pre-eclampsia [14, 15]. Urine testing also facilitates decisions to start ANC through early diagnosis of pregnancy [16]. ANC tetanus vaccination injections help prevent the fatal tetanus among women and their babies. Blood sample provision helps in screening and prompt management of anemia which can lead to low birth weight and maternal mortality [17]. Maternal infections detrimental to the woman and her unborn baby like malaria, hepatitis B, syphilis, HIV, gonorrhea and chlamydia are identified early during pregnancy through provision of a blood sample [18–22]. HIV testing during antenatal care, often done through blood samples is an entry point to elimination of mother to child HIV transmission (eMTCT), prevention and treatment for the partner and community at large. Provision of deworming treatment helps treat maternal infections that also have effects on the unborn baby, especially in helminths endemic areas [23]. Iron and folic acid supplementation is key in preventing anemia, including adverse effects of postpartum hemorrhage, preterm births and low birth weight babies [24]. Some components of ANC like counseling help improve early initiation of exclusive breast feeding, uptake of childhood immunization and attendance of postnatal care.

Women in hard to reach geographically isolated locations like island fishing communities (FCs) on Lake Victoria, may be at increased risk of maternal death due to inadequate or no provision of care components during ANC visits [25]. These island fishing communities are considered.

hard to reach, resource limited settings, due to maritime challenges and rural location.

Kalangala district has 64 habitable islands FCs with only 17 health facilities (mainly level II and III) serving these islands, of which none is a hospital [26]. There is poor geographic accessibility across one island to another with a health facility, often the nearest health facility being 8–12 km away. The cost of transporting a mother who needs ANC and delivery is about 55 US dollars, which includes hiring a boat with an engine, a coxswain and buying fuel for the trip [27]. Kalangala district also has a shortage of skilled birth attendants (Doctor, Nurse and Midwife), with only 16.4 skilled birth attendants per 10,000 people, still far below WHO target for sustainable development [26, 28, 29].

We assessed the components of care received by women in these island fishing communities, to inform antenatal care services provision among these hard to reach settings.

## Methods

### Study design aim and setting

This community based cross-sectional survey aimed at characterizing components of care received by ANC attendees living in 6 rural islands (Buwuvu, Jaana, Kitobo, Lulamba, Namisoke and Sserinya) of Kalangala district, Uganda. The islands were selected from 12 islands where the authors had previous research experience based on remoteness (inaccessibility), with the nearest being 2 h by motorized boat ride from Entebbe mainland and having a population of at least 1000 people [29]. The survey was conducted during January–May 2018, using a questionnaire developed for this study with questions from DHS Program surveys [30]. See questionnaire under supplementary material. Further details of the methods are described elsewhere [31].

Women who attended ANC were asked if they received the following seven components of ANC at least once: 1) blood pressure measurement, 2) provision of a blood sample, 3) provision of a urine sample, 4) tetanus vaccination, 5) IPTp including number of times, 6) deworming treatment, and 7) iron-folic acid supplements.

We did not ask women about receipt of any form of counseling. Women were also not asked how many times they received each ANC component, or at what months of pregnancy or with which type of health worker or at what health facility.

### Statistical methods

This analysis aimed at answering the following questions;

1. What proportion of women who attended care received all seven components as part of the Uganda guidelines goal-oriented ANC protocol?

2. What factors are associated with receipt of all seven components of care among women who attended ANC?

#### Study variables

The dependent variable was receipt of all seven ANC components by women who attended care during the most recent or current pregnancy. Receipt of all ANC components was generated as a binary outcome with “No, all ANC components” for women who did not receive all seven components at least once and “Yes, all ANC components” for women who received all seven components at least once.

The independent variables included; age, tribal affiliation, highest education attained, women’s occupation, partner occupation, total births by a woman, reported pregnancy status at interview, timing of first ANC visit, ANC facility location (island vs mainland), provider cadre and visits attended (less than four or at least four). Frequency tabulation of women’s characteristics by receipt of all ANC components was used to analyze the distribution of study related factors and relationships with the dependent variable. Grouping of independent variables was based on their logical relationship with the dependent variable (receipt of all seven ANC components) at bivariable analysis. Bivariable chi-square tests were used to assess the associations between independent variables and the dependent variable at 95% significance level.

Multivariable logistic regression modeling was used to determine participant characteristics associated with receipt of all ANC components by women who attended ANC at least once. Selection of predictor variables included for the multivariable model was based on previous literature, biological plausibility or statistical significance (*P*-value ≤0.2) at bivariable analysis. We selected a priori women’s age (binary), occupation (binary), ethnicity (binary), duration of stay in study community (binary), total births (categorical), number of ANC times (counts), cadre of ANC provider (binary) and location of ANC service (binary) as independent variables for the multivariable model. We assessed for collinearity and removed variables that did not improve the model or were highly correlated with other variables in the model, with the final best suited predictors in the model having the lowest *P*-values (up to *p* = 0.05), lowest model Akaike’s information criterion (AIC) and Bayesian information criterion (BIC) values. Adjusted Odds ratios (aOR), *P*-values and 95% confidence intervals (CI) were used to report associations. All analyses were done using STATA® version 15.1 [32]. Tables were created using asdoc [33].

## Results

### Participants characteristics

The survey involved 486 women from 6 island FCs and was conducted between January and May 2018, 246 (50.6%) of whom were currently pregnant and 240 (49.4%) were not currently pregnant but had been within the past 6 months. Women’s age ranged between 15 to 45 years, majority were married (87.0%), their main occupation was housewife (stay-at-home mums) (45.1%) and never studied beyond 7 years of education (69.1%). See Table 1.

**Table 1:** Characteristics of participating women

Characteristics	Frequency	Percentage
Age groups (Years)	(*n* = 486)	
15–24	193	39.7
25–49	293	60.3
Age at first pregnancy (Years)	(*n* = 486)	
< 15	52	10.7
15–19	350	72.0
≥ 20	84	17.3
Marital status	(*n* = 486)	
Married	423	87.0
Not married	63	13.0
Tribal affiliation	(*n* = 486)	
None Baganda	276	56.8
Baganda	210	43.2
Highest education completed (Years)	(*n* = 486)	
≥ 8 (8th grade and above)	150	30.9
1–7 (up to 7th grade)	304	62.5
0 (None)	32	6.6
Occupation group	(*n* = 486)	
Agriculture	43	8.9
Housewife	219	45.1
Bar, Restaurant, lodge worker/owner	58	11.9
Fishing related	42	8.6
Others	124	25.5
Partner occupation	(*n* = 423)	
Fishing related	299	70.7
None fishing related	124	29.3
Duration of community stay	(*n* = 486)	
3-11 months	94	19.3
≥ 1 year	392	80.7
Total births (median = 3, range 0–11)	(*n* = 486)	
None	46	9.5
1–3	287	59.0
≥ 4	153	31.5
Any ANC attendance	(*n* = 486)	
No attendance	170	35.0
Attended	316	65.0
First ANC visit timing	(*n* = 316)	
≤ 3 months pregnant	118	37.3
> 3 months pregnant	198	62.7
ANC provider	(*n* = 316)	
Nurse/Midwife	296	93.7
Doctor	20	6.3
ANC location	(*n* = 316)	
Islands	247	78.2
Mainland	69	21.8
ANC components received (median = 6)	(*n* = 316)	
Iron supplementation	308	97.5
Blood sample	281	88.9
T.T injection	275	87.0
Blood pressure measured	266	84.2
IPTp	261	82.6
Deworming	222	70.3
Urine sample	106	33.5
Last HIV test period	(*n* = 486)	
Within 3 months	302	62.1
Over 3 months	127	26.1
Never tested in life	1	0.2
On ART	56	11.5
Received all seven ANC components	(*n* = 316)	
No	258	82.0
Yes	58	18.4

Total pregnancies by each woman ranged from 1 to 11, a third (32.9%) had five or more. Median parity was 3, ranging from 0 to 11, with nearly a third (31.5%) having at least four births.

All 486 women reported they would have liked being attended to by a skilled ANC provider during current or recent pregnancy. Over three fifths (65.0%) had at least one ANC visit during current or most recent pregnancy, with majority (62.7%) starting late after the first trimester (mean/SD months at start =3.96/1.65) and attending from island-based health facilities (78.2%). See Table 1.

### Components of ANC

Less than one in five women who attended at least one visit received all seven assessed ANC components (18.4%). Nearly all women received at least one component (99.7%), with almost a third (28.5%) and two fifths (36.1%) receiving five and six components respectively. The most common ANC component received was iron supplementation with the least common being urine checks. Among women who had received three ANC components, they were often iron supplementation, blood check and tetanus vaccination injection. See Table 1.

Even among those who reported attending four or more ANC visits, few (23.6%) received all the seven components of ANC. Just a fifth (20.3%) of women who started care early within the first 3 months of pregnancy received all ANC components. See Table 2. More women aged 25–49 years received all ANC components relative to those aged 15–24 years at the time of the survey (22.0% vs 12.8%, *P* < 0.05). More ANC attendees affiliated to the Baganda tribe received all care components compared to none-Baganda (23.7% vs 13.6%, *P* < 0.05). Fewer stay-at-home mums (Housewives) received all ANC components compared to those who were not stay-at-home moms (13.4% vs 22.8%, *P* < 0.05). Pregnancy status at the time of the interview was not associated with receipt of all the seven ANC components (currently pregnant [13.5%], birth within 6 months [22.6%], abortion or miscarriage within 6 months [13.6%], *p* = 0.11). A higher proportion of women who attended ANC from mainland-based facilities received all care components relative to those who attended islands-based health facilities (27.5% vs 15.8%, *P* < 0.05). Relative to those who received care from Nurses or Midwives, more women attended to by Doctors received all ANC components (16.6% vs 45.0%, *P* < 0.05). See Table 2.

**Table 2:** ANC attendees’ characteristics by components receipt (*n* = 316)

Characteristics	Did not receive all ANC components	Received all ANC components	*P*-value
	n (%)	n (%)	
Age groups			< 0.05
15–24	109 (87.2)	16 (12.8)	
25–49	149 (78.0)	42 (22.0)	
Highest education completed (Years)			0.65
0 (None)	18 (85.7)	3 (14.3)	
1–7 (1st-7th grade)	163 (82.7)	34 (17.3)	
≥ 8 (8th grade and above)	77 (78.6)	21 (21.4)	
Tribal affiliation			< 0.05
None Baganda	153 (86.4)	24 (13.6)	
Baganda	105 (75.5)	34 (24.5)	
Occupation			< 0.05
Housewife	129 (86.6)	20 (13.4)	
None-Housewife	129 (77.3)	38 (22.7)	
Partner occupation	(*n* = 277)		0.07
Fishing related	162 (84.4)	30 (15.6)	
None Fishing related	64 (75.3)	21 (24.7)	
Pregnancy status at interview			0.11
Childbirth within 6 months	130 (77.4)	38 (22.6)	
Currently pregnant	109 (86.5)	17 (13.5)	
Loss pregnancy within 6 months	19 (86.4)	3 (13.6)	
ANC location			< 0.05
Islands	208 (84.2)	39 (15.8)	
Mainland	50 (72.5)	19 (27.5)	
ANC provider			< 0.05
Nurse/Midwife	247 (83.4)	49 (16.6)	
Doctor	11 (55.0)	9 (45.0)	
First ANC visit timing			0.48
≤ 3 months pregnant	94 (79.7)	24 (20.3)	
> 3 months pregnant	164 (82.8)	34 (17.2)	
ANC visits attended			< 0.05
< Four	151 (85.8)	25 (14.2)	
≥ Four	107 (76.4)	33 (23.6)	
Total births			0.10
None	25 (96.2)	1 (3.8)	
1–3	145 (79.2)	38 (20.8)	
≥ 4	88 (82.2)	19 (17.8)	

In our multivariable modeling, we found that women who attended care from mainland facilities were twice as likely to have received all ANC components as those who received care from island facilities (aOR = 1.8; 95% CI:0.9–3.7). Women who reported being attended to by a Doctor were thrice as likely to have received all ANC components as those seen by a Midwife/Nurse (aOR = 3.2; 95% CI:1.1–9.1). The number of times women attended ANC was associated with likelihood of having received all components of care (aOR = 1.3, 95% CI: 1.1–1.5). Older women were more likely to have received all seven components compared to younger women (ages 25+ compared to < 25 years, aOR = 2.0, 95% CI: 1.0–3.9), and Baganda tribe was also associated with increased odds of receiving the full package. See Table 3.

**Table 3:** Crude (cOR) and adjusted odds ratios (aOR) of factors associated with all ANC components receipt among clinic attendees (*n* = 316)

All components receipt	cOR	[95% Conf.Interval]	aOR	[95% Conf.Interval]
ANC provider				
Nurse/Midwife	(ref)		(ref)	
Doctor	4.1	1.6 10.5	3.2	1.1 9.1
ANC times	1.3	1.1 1.5	1.3	1.1 1.5
ANC location				
Island	(ref)		(ref)	
Mainland	2.0	1.1 3.8	1.8	0.9 3.7
Tribal affiliation				
Non-Baganda	(ref)		(ref)	
Baganda	2.0	1.2 3.7	2.1	1.1 3. 8
Age group (Years)				
15–24	(ref)		(ref)	
25–49	1.9	1.0 3.6	2.0	1.0 3.9

*** *p < 0.001,* ** *p < 0.01,* * *p ≤ 0.05*

## Discussion

Proper ANC is key to improving reproductive health, increasing skilled birth attendance, reducing maternal morbidity and mortality. We observed that women who attend ANC clinics often did not receive the recommended components of care, with only 18.4% of those who attended at least once reporting receiving all seven assessed components as by Ugandan government guidelines [7]. Less than a quarter of women who managed to attend four visits did not receive all seven components at least once. Whilst many women attended ANC at least once or four times, comparatively fewer women received all seven ANC components during those visits. This highlights the need to emphasize and assess receipt of components of care during routine monitoring of ANC quality processes in these communities.

Notwithstanding efforts encouraging pregnant women to start ANC early, a large proportion of those who started within the first 3 months of pregnancy did not receive all components, yet it is expected they receive all earlier during ANC attendance [7]. This dampens efforts towards improving maternal health as opportunities to identify, prevent and manage complications through receipt of all ANC components are missed. HIV burden is high in these FCs [34], eMTCT including other early treatment and care interventions are likely to be less effective when started late in pregnancy.

An important public health finding in these island FCs is that mainland health facilities provided more complete ANC relative to those on the islands. Islands are rural and hard to reach, which may impact on the quality of ANC, as supplies and equipment might have been inadequate or lacking [2, 35]. Though unlikely, skilled attendants might have not provided these components of care despite the equipment and supplies being available due to lack of motivation or heavy workload. We did not assess availability of supplies or workload of staff at the facilities where women received ANC. Island based facilities are also more likely to employ poorly skilled ANC attendants, as few highly skilled health workers want to work in these hard-to-reach, rural FCs. All our study participants were residents of remote islands, and women who attended ANC from mainland health facilities might have had better access to maternal health information and financial means to seek all components of care at facilities where they were easily provided. This adds to previous work on rural-urban ANC differences, uniquely highlighting differences between mainland and islands FCs [36–38].

The cadre of skilled attendant was associated with receipt of all seven ANC components, as women who reported seeing a Doctor were likely to have received all care components relative to those who received care from Nurses or Midwives. Similar to previous work in other settings [39], skilled attendants have a big role during ANC in these communities.

Attendance of more visits was associated with a higher likelihood of receiving all seven ANC components, as others have observed [40, 41]. Women who attend more visits increase chances of receiving components that were missed during the preceding visits, especially if the lack of receipt was due to stock outs of supplies or faulty equipment that would be later repaired. Women 25 years or older were more likely to have received all ANC components than their younger counterparts, perhaps as they had prior experiences with the health care system and might have known the importance of receiving all components of care. This adds to similar to work in India [42], were older women received more adequate care than adolescents.

Urine testing was the least received component, similar to the results from another ANC survey in Uganda [37]. The low receipt of urine testing may be that it requires more technical expertise with a functional laboratory which may not be readily available at ANC facilities in these island communities [2], or that the facilities and/ or supplies for collecting urine are inadequate. Infrequent refresher training of providers and poor adherence to standards [43], may also have contributed. This undesirably affects diagnosis and management of hypertensive disorders of pregnancy, urinary tract infections and gestational diabetes in these remote communities, where prompt referrals to the mainland is a challenge for care not available on the islands. There is need to improve the provision of urine testing, to promptly manage pregnancy conditions and improve ANC quality.

A limitation of this study was the lack of comparison of self-reports to medical records as ANC attendance occurred from diverse locations. We were unable to visit ANC centers to assess health care provider perspectives, review stock outs, training, and ANC center-reported administration of the seven ANC components. Recall bias, a challenge with self-reports, was minimized using a shorter duration of recall (6 months) for most of the data we collected.

It is also possible that women who had complicated pregnancies led to over reporting of all ANC components receipt, as we did not assess the frequency of complicated pregnancies. Despite the limitations, these findings highlight crucial health services propositions for ANC in these islands.

## Conclusions

The study shows that receipt of all ANC components was meagre in these islands FCs relative to the mainland. Wide differences existed between number of visits attended and receipt of all ANC components. Cost effective endeavors to improve components of care received by ANC attendees are urgently needed.

Task shifting of less technical components of care to CHWs may help improve receipt of all ANC components amidst critical shortages of skilled health workers in these island communities. Implementation programs should consider assessing components of care received by women attending ANC in these hard to reach FCs.

## Supplementary information

**Additional file 1.**

## Data Availability

The dataset used and analyzed during the current study is readily available from the corresponding author upon reasonable request.

## Abbreviations

AIC: Akaike’s information criterion
ANC: Antenatal care
aOR: Adjusted odds ratio
BIC: Bayesian information criterion
CHW: Community health worker
CI: Confidence interval
cOR: Crude odds ratio
eMTCT: Elimination of mother to child transmission of HIV
FCs: Fishing communities
FWA: Federal wide assurance
HIV: Human immunodeficiency virus
IAVI: International AIDS vaccine initiative
ICRH: International center for reproductive health
IPTp: Intermittent preventive treatment with sulphadoxine/pyrimethamine
ODK: Open data kit
OR: Odds ratio
SD: Standard deviation
STI: Sexually transmitted infection
UVRI: Uganda virus research institute
WHO: World health organization
